# *trans*-Dichloridobis[dicyclo­hex­yl(phen­yl)phosphane-κ*P*]palladium(II)

**DOI:** 10.1107/S1600536812010100

**Published:** 2012-03-10

**Authors:** Andrew R. Burgoyne, Reinout Meijboom, Hezron Ogutu

**Affiliations:** a Research Centre for Synthesis and Catalysis, Department of Chemistry, University of Johannesburg, PO Box 524 Auckland Park, Johannesburg 2006, South Africa

## Abstract

The title compound, [PdCl_2_{P(C_6_H_11_)_2_(C_6_H_5_)}_2_], forms a monomeric complex with a *trans*-square-planar geometry. The Pd—P bond lengths are 2.3343 (5) Å, as the Pd atom lies on an inversion centre, while the Pd—Cl bond lengths are 2.3017 (4) Å. The observed structure was found to be closely related to [PdCl_2_{P(C_6_H_11_)_3_}_2_] [Grushin *et al.* (1994 ▶). *Inorg. Chem.* **33**, 4804–4806], [PdBr_2_{P(C_6_H_11_)_3_}_2_] [Clarke *et al.* (2003 ▶). *Dalton Trans.* pp. 4393–4394] and [PdCl_2_P(C_6_H_11_)_2_(C_7_H_7_)}_2_] [Vuoti *et al.* (2008 ▶). *Eur. J. Inorg. Chem.* pp. 397–407] (C_6_H_11_ is cyclo­hexyl and C_7_H_7_ is *o*-tol­yl). One of the cyclo­hexyl rings is disordered with the phenyl ring in a 0.587 (9):413 (9) ratio. Five long-range C—H⋯Cl inter­actions were observed within the structure.

## Related literature

For a review on related compounds, see: Spessard & Miessler (1996 ▶). For the synthesis of the starting materials, see: Drew & Doyle (1990 ▶). For similar *R*-P_2_PdCl_2_ compounds, see: Ogutu & Meijboom (2011 ▶); Muller & Meijboom (2010*a* ▶,*b* ▶). For their applications, see: Bedford *et al.* (2004 ▶).
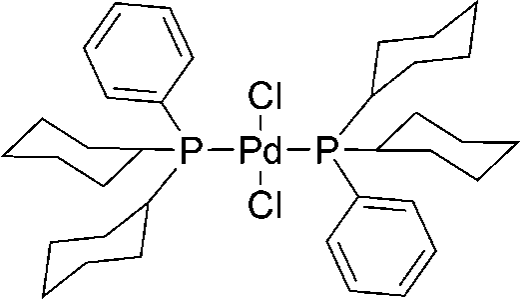


## Experimental

### 

#### Crystal data

[PdCl_2_(C_18_H_27_P)_2_]
*M* *_r_* = 726.03
Triclinic, 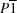

*a* = 9.439 (4) Å
*b* = 10.095 (4) Å
*c* = 10.623 (5) Å
α = 113.115 (2)°
β = 107.321 (2)°
γ = 91.625 (2)°
*V* = 876.5 (7) Å^3^
*Z* = 1
Mo *K*α radiation
μ = 0.80 mm^−1^
*T* = 100 K
0.27 × 0.13 × 0.11 mm

#### Data collection

Bruker X8 APEXII 4K KappaCCD diffractometer
Absorption correction: multi-scan (*SADABS*; Bruker, 2007 ▶) *T*_min_ = 0.885, *T*_max_ = 0.918
12144 measured reflections
2931 independent reflections
2891 reflections with *I* > 2σ(*I*)
*R*_int_ = 0.024

#### Refinement

*R*[*F* ^2^ > 2σ(*F* ^2^)] = 0.021
*wR*(*F* ^2^) = 0.051
*S* = 1.19
2931 reflections
266 parameters
12 restraints
H-atom parameters constrained
Δρ_max_ = 0.37 e Å^−3^
Δρ_min_ = −0.38 e Å^−3^

### 

Data collection: *APEX2* (Bruker, 2007 ▶); cell refinement: *SAINT-Plus* (Bruker, 2007 ▶); data reduction: *SAINT-Plus* and *XPREP* (Bruker, 2007 ▶); program(s) used to solve structure: *SHELXS97* (Sheldrick, 2008 ▶); program(s) used to refine structure: *SHELXL97* (Sheldrick, 2008 ▶); molecular graphics: *DIAMOND* (Brandenburg & Putz, 2005 ▶); software used to prepare material for publication: *WinGX* (Farrugia, 1999 ▶).

## Supplementary Material

Crystal structure: contains datablock(s) I, global. DOI: 10.1107/S1600536812010100/zl2455sup1.cif

Structure factors: contains datablock(s) I. DOI: 10.1107/S1600536812010100/zl2455Isup2.hkl

Additional supplementary materials:  crystallographic information; 3D view; checkCIF report

## Figures and Tables

**Table 1:** Hydrogen-bond geometry (Å, °)

*D*—H⋯*A*	*D*—H	H⋯*A*	*D*⋯*A*	*D*—H⋯*A*
C8—H8*B*⋯Cl1	0.97	2.91	3.559 (2)	125
C5—H5⋯Cl1^i^	0.93	2.94	3.619 (7)	131
C13—H13⋯Cl1^ii^	0.98	2.68	3.254 (11)	118
C18—H18*B*⋯Cl1^ii^	0.97	2.97	3.542 (13)	119
C15—H15*A*⋯Cl1^iii^	0.97	3.02	3.800 (8)	139

Symmetry codes: (i) 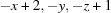; (ii) 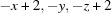; (iii) 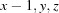.

**Fig. 1.**
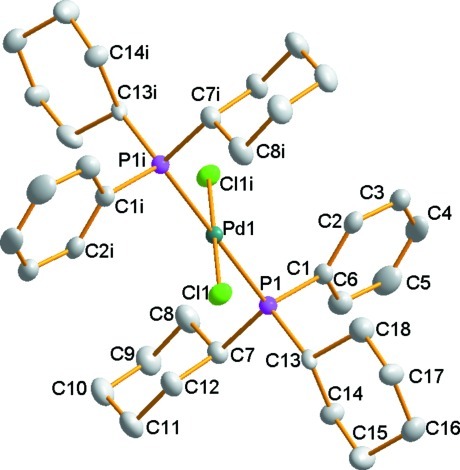
The structure of the trans-dichlorobis (dicyclohexylphenylphosphine)palladium(II) showing 50% probability displacement ellipsoids. Symmetry code to generate molecule through inversiont point: (iv) 2 - x, -y, 2 - z. Disordered and hydrogen atoms were omitted for clarity.

**Fig. 2.**
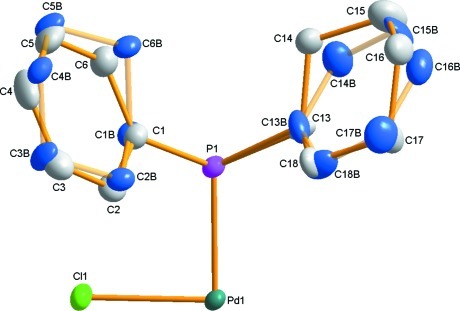
The structure of the disordered cyclohexyl and phenyl rings in trans-dichlorobis(dicyclohexylphenylphosphine)palladium(II), with the lower occupancy atoms shown in blue.

## supplementary crystallographic information

### Comment

Complexes involving palladium metal centres are amongst some of the most popular catalytic precursors in organic synthesis due to their catalytic abilities. They are used in carbon-carbon bond formation reactions like the Heck, Stille and Suzuki reactions (Bedford *et al.*, 2004).

[PdCl_2_(*L*)_2_] (*L* = tertiary phosphine, arsine or stibine) complexes can conveniently be prepared by the substitution of 1,5-cyclooctadiene (COD) from [PdCl_2_(COD)]. The title compound, *trans*-[PdCl_2_(C_18_H_27_P)_2_], crystallizes with the Pd atom on a center of symmetry and each pair of equivalent ligands in a mutually *trans* orientation. The geometry is, therefore, slightly distorted square planar and the Pd atom is not elevated out of the coordinating atom plane. All angles in the coordination polyhedron are close to the ideal value of 90°, with P—Pd—Cl = 89.296 (16)° and P—Pd—Cl^i^ = 90.704 (16)°. As required by the crystallographic symmetry, the P—Pd—P^i^ and Cl—Pd—Cl^i^ angles are 180°. The symmetry code used to define atoms through the inversion point is: (iv) 2 - *x*, -*y*, 2 - *z*.

One of the cyclohexyl rings, C13–C18, in the title compound is disordered with the phenyl ring, C1–C6, over the same positions in a 59:41 (9) occupancy ratio.

The title compound compares well with other closely related Pd^II^ complexes from the literature containing two chloro and two tertiary phosphine ligands in a *trans* geometry (Muller & Meijboom, 2010*a*, *b*). The title compound, having a Pd—Cl bond length of 2.3017 (4) Å and a Pd—P bond length of 2.3343 (5) Å, fits well into the typical range for complexes of this kind. Notably the title compound did not crystallize as a solvated complex; these type of Pd^II^ complexes have a tendency to crystallize as solvates (Ogutu & Meijboom, 2011).

Due to the disorder of the cyclohexyl ring and phenyl ring, the crystilline structure for the title compound forms an isostructure with a variety of [PdCl_2_(P*R*_3_)_2_] compounds (*R* = any combination of aryl and cylcohexyl rings). Notably, the title compound is quintessentially isostructural with: [PdCl_2_{P(C_6_H_11_)_3_}_2_] (Grushin *et al.*, 1994); [PdBr_2_{P(C_6_H_11_)_3_}_2_] (Clarke *et al.*, 2003); and [PdCl_2_{P(C_6_H_11_)_2_(C_7_H_7_)}_2_] (Vuoti *et al.*, 2008) ((C_6_H_11_) = cyclohexyl, (C_7_H_7_) = *o*-tolyl). The Pd–P and Pd–*X* (*X* = Br and Cl) bond lengths were compared and it was observed that they were all within the same range of 2.3–2.4 Å. The angles between the bonds around the Pd atom were all observed to be approximately right angles.

A weak hydrogen bond exists between C13—H13···Cl1^i^ (Symmetry code: -*x* + 2, -*y*, -*z* + 2) with the distance listed in Table 1. Four other longer range hydrogen interactions exist as shown in Table 1.

### Experimental

Dicyclohexylphenylphosphine (0.05 g, 0.35 mmol) was dissolved in acetone (5 cm^3^). A solution of [Pd(COD)Cl_2_] (0.05 g, 0.17 mmol) in acetone (5 cm^3^) was added to the phosphine solution. The mixture was stirred for 5 minutes, after which the solution was left to crystallize. Yellow crystals of the title compound were obtained. ^1^H NMR (CDCl_3_, 400 MHz, p.p.m.): 7.6–7.5 (*m*, 4H), 7.4 (*m*, 6H), 2.6 (*t*, 4H), 2.1 (*d*, 4H), 1.8–1.7 (*m*, 10H), 1.4–1.2 (*m*, 20H), 1.1–1.0 (*m*, 6H). ^31^P{H} NMR (CDCl_3_, 162.0 MHz, p.p.m.): 28.05. IR (cm^-1^): 2925, 2849, 2161, 2023, 1977, 1446, 1433, 1261, 1109, 1011, 848, 773, 700 and 690.

### Refinement

The undisordered quintessential cyclohexyl ring, C7–C12, was used to model the disordered cyclohexyl ring, C1B–C6B, by restraining the two rings to have similar bond lengths and 1,3 atom distances within a standard deviation of 0.02 Å. (SAME command in Shelxtl, Sheldrick, 2008). Atoms C1 and C1B, the two *ipso–*carbons for the disordered phenyl and dicyclohexyl rings, were constrained to have identical ADPs. The phenyl ring has been constrained to resemble an ideal hexagon with C—C distances of 1.39 Å All hydrogen atoms were positioned geometrically with C—H = 0.98 Å for H atoms bonded to tertiary C atoms, 0.97 Å for methylene H atoms, and 0.93 Å for aromatic H atoms. . All hydrogen atoms were allowed to ride on their parent atoms with *U*_iso_(H) = 1.2*U*_eq_(C). The remaining highest electron peak was 0.37 at 0.95 Å from P1 and the deepest hole was -0.38 at 0.92 Å from Pd1.

### Crystal data

**Table:**

[PdCl_2_(C_18_H_27_P)_2_]	*Z* = 1
*M**_r_* = 726.03	*F*(000) = 380
Triclinic, *P*1	*D*_x_ = 1.375 Mg m^−^^3^
Hall symbol: -P 1	Mo *K*α radiation, λ = 0.71069 Å
*a* = 9.439 (4) Å	Cell parameters from 9929 reflections
*b* = 10.095 (4) Å	θ = 2.2–24.9°
*c* = 10.623 (5) Å	µ = 0.80 mm^−^^1^
α = 113.115 (2)°	*T* = 100 K
β = 107.321 (2)°	Conical, yellow
γ = 91.625 (2)°	0.27 × 0.13 × 0.11 mm
*V* = 876.5 (7) Å^3^	

### Data collection

**Table:**

Bruker X8 APEXII 4K KappaCCD diffractometer	2931 independent reflections
Radiation source: fine-focus sealed tube	2891 reflections with *I* > 2σ(*I*)
Graphite monochromator	*R*_int_ = 0.024
φ and ω scans	θ_max_ = 24.9°, θ_min_ = 2.2°
Absorption correction: multi-scan (*SADABS*; Bruker, 2007)	*h* = −10→11
*T*_min_ = 0.885, *T*_max_ = 0.918	*k* = −11→11
12144 measured reflections	*l* = −11→12

### Refinement

**Table:**

Refinement on *F*^2^	Primary atom site location: structure-invariant direct methods
Least-squares matrix: full	Secondary atom site location: difference Fourier map
*R*[*F*^2^ > 2σ(*F*^2^)] = 0.021	Hydrogen site location: inferred from neighbouring sites
*wR*(*F*^2^) = 0.051	H-atom parameters constrained
*S* = 1.19	*w* = 1/[σ^2^(*F*_o_^2^) + (0.0125*P*)^2^ + 0.5911*P*] where *P* = (*F*_o_^2^ + 2*F*_c_^2^)/3
2931 reflections	(Δ/σ)_max_ = 0.001
266 parameters	Δρ_max_ = 0.37 e Å^−^^3^
12 restraints	Δρ_min_ = −0.38 e Å^−^^3^

### Special details

**Table:**

Experimental. The intensity data was collected on a Bruker X8 Apex II 4 K Kappa CCD diffractometer using an exposure time of 10 s/frame. A collection frame width of 0.5° covering up to θ = 24.9° resulted in 97% completeness accomplished.
Geometry. All esds (except the esd in the dihedral angle between two l.s. planes) are estimated using the full covariance matrix. The cell esds are taken into account individually in the estimation of esds in distances, angles and torsion angles; correlations between esds in cell parameters are only used when they are defined by crystal symmetry. An approximate (isotropic) treatment of cell esds is used for estimating esds involving l.s. planes.
Refinement. Refinement of F^2^ against ALL reflections. The weighted R-factor wR and goodness of fit S are based on F^2^, conventional R-factors R are based on F, with F set to zero for negative F^2^. The threshold expression of F^2^ > 2sigma(F^2^) is used only for calculating R-factors(gt) etc. and is not relevant to the choice of reflections for refinement. R-factors based on F^2^ are statistically about twice as large as those based on F, and R- factors based on ALL data will be even larger.

### Fractional atomic coordinates and isotropic or equivalent isotropic displacement parameters (Å^2^)

**Table:**

	*x*	*y*	*z*	*U*_iso_*/*U*_eq_	Occ. (<1)
C1	0.8288 (11)	−0.1156 (10)	0.6237 (7)	0.0215 (7)	0.587 (9)
C2	0.9177 (12)	−0.2245 (12)	0.6081 (9)	0.025 (3)	0.587 (9)
H2	0.9665	−0.2441	0.6874	0.030*	0.587 (9)
C3	0.9336 (9)	−0.3042 (9)	0.4741 (10)	0.030 (3)	0.587 (9)
H3	0.9931	−0.3770	0.4637	0.036*	0.587 (9)
C4	0.8606 (8)	−0.2749 (8)	0.3556 (8)	0.038 (2)	0.587 (9)
H4	0.8713	−0.3282	0.2660	0.045*	0.587 (9)
C5	0.7718 (9)	−0.1660 (9)	0.3712 (7)	0.040 (4)	0.587 (9)
H5	0.7229	−0.1465	0.2920	0.048*	0.587 (9)
C6	0.7558 (7)	−0.0864 (7)	0.5053 (8)	0.0294 (15)	0.587 (9)
H6	0.6964	−0.0135	0.5157	0.035*	0.587 (9)
C1B	0.8337 (16)	−0.1023 (17)	0.6235 (11)	0.0215 (7)	0.413 (9)
H1B	0.9155	−0.0324	0.6342	0.026*	0.413 (9)
C2B	0.892 (2)	−0.2484 (19)	0.5907 (14)	0.021 (3)	0.413 (9)
H2BA	0.8132	−0.3233	0.5748	0.025*	0.413 (9)
H2BB	0.9771	−0.2397	0.6732	0.025*	0.413 (9)
C3B	0.9388 (17)	−0.2926 (17)	0.4549 (15)	0.027 (3)	0.413 (9)
H3BA	1.0238	−0.2223	0.4747	0.033*	0.413 (9)
H3BB	0.9705	−0.3873	0.4327	0.033*	0.413 (9)
C4B	0.8129 (12)	−0.2994 (11)	0.3271 (10)	0.023 (2)	0.413 (9)
H4BA	0.7318	−0.3765	0.3012	0.027*	0.413 (9)
H4BB	0.8477	−0.3228	0.2448	0.027*	0.413 (9)
C5B	0.7549 (14)	−0.1569 (12)	0.3589 (11)	0.023 (3)	0.413 (9)
H5BA	0.6712	−0.1655	0.2751	0.028*	0.413 (9)
H5BB	0.8337	−0.0811	0.3773	0.028*	0.413 (9)
C6B	0.7032 (10)	−0.1141 (10)	0.4917 (11)	0.0224 (18)	0.413 (9)
H6BA	0.6677	−0.0212	0.5118	0.027*	0.413 (9)
H6BB	0.6207	−0.1871	0.4718	0.027*	0.413 (9)
C7	0.7420 (2)	0.1499 (2)	0.8114 (2)	0.0247 (4)	
H7	0.6498	0.1300	0.7292	0.030*	
C8	0.8584 (3)	0.2478 (2)	0.8001 (2)	0.0335 (5)	
H8A	0.8772	0.1976	0.7093	0.040*	
H8B	0.9522	0.2690	0.8792	0.040*	
C9	0.8040 (3)	0.3903 (2)	0.8064 (2)	0.0355 (5)	
H9A	0.8816	0.4529	0.8034	0.043*	
H9B	0.7152	0.3697	0.7224	0.043*	
C10	0.7666 (3)	0.4687 (2)	0.9438 (2)	0.0353 (5)	
H10A	0.8579	0.4988	1.0275	0.042*	
H10B	0.7267	0.5558	0.9424	0.042*	
C11	0.6531 (3)	0.3725 (2)	0.9585 (3)	0.0365 (5)	
H11A	0.5577	0.3531	0.8818	0.044*	
H11B	0.6378	0.4236	1.0508	0.044*	
C12	0.7038 (2)	0.2278 (2)	0.9498 (2)	0.0288 (4)	
H12A	0.6242	0.1658	0.9509	0.035*	
H12B	0.7916	0.2459	1.0339	0.035*	
C13	0.6240 (7)	−0.1337 (11)	0.7724 (14)	0.017 (2)	0.587 (9)
H13	0.6085	−0.0958	0.8667	0.020*	0.587 (9)
C14	0.4830 (6)	−0.1221 (6)	0.6629 (6)	0.0260 (12)	0.587 (9)
H14A	0.4726	−0.0202	0.6898	0.031*	0.587 (9)
H14B	0.4928	−0.1611	0.5672	0.031*	0.587 (9)
C15	0.3432 (8)	−0.2058 (7)	0.6579 (13)	0.038 (2)	0.587 (9)
H15A	0.3289	−0.1617	0.7515	0.046*	0.587 (9)
H15B	0.2557	−0.1994	0.5855	0.046*	0.587 (9)
C16	0.3565 (8)	−0.3654 (7)	0.6210 (8)	0.0311 (14)	0.587 (9)
H16A	0.2694	−0.4143	0.6255	0.037*	0.587 (9)
H16B	0.3591	−0.4128	0.5228	0.037*	0.587 (9)
C17	0.4985 (10)	−0.3777 (10)	0.7268 (16)	0.029 (2)	0.587 (9)
H17A	0.5083	−0.4798	0.6985	0.035*	0.587 (9)
H17B	0.4903	−0.3397	0.8232	0.035*	0.587 (9)
C18	0.6381 (8)	−0.2948 (9)	0.7318 (17)	0.023 (3)	0.587 (9)
H18A	0.6513	−0.3373	0.6377	0.028*	0.587 (9)
H18B	0.7258	−0.3027	0.8029	0.028*	0.587 (9)
C13B	0.6193 (7)	−0.1534 (11)	0.7533 (16)	0.027 (5)	0.413 (9)
C14B	0.4787 (8)	−0.1111 (7)	0.7229 (11)	0.0331 (19)	0.413 (9)
H14C	0.4707	−0.0176	0.7283	0.040*	0.413 (9)
C15B	0.3501 (7)	−0.2087 (9)	0.6846 (13)	0.045 (5)	0.413 (9)
H15C	0.2560	−0.1804	0.6642	0.054*	0.413 (9)
C16B	0.3620 (11)	−0.3485 (8)	0.6766 (10)	0.033 (2)	0.413 (9)
H16C	0.2760	−0.4137	0.6509	0.040*	0.413 (9)
C17B	0.5026 (14)	−0.3907 (9)	0.7070 (17)	0.035 (5)	0.413 (9)
H17C	0.5106	−0.4843	0.7016	0.042*	0.413 (9)
C18B	0.6312 (10)	−0.2932 (14)	0.745 (2)	0.032 (6)	0.413 (9)
H18C	0.7253	−0.3215	0.7657	0.038*	0.413 (9)
P1	0.79648 (5)	−0.02795 (5)	0.79688 (5)	0.01688 (11)	
Cl1	1.15978 (5)	0.06458 (5)	0.89925 (5)	0.02496 (11)	
Pd1	1.0000	0.0000	1.0000	0.01502 (8)	

### Atomic displacement parameters (Å^2^)

**Table:**

	*U*^11^	*U*^22^	*U*^33^	*U*^12^	*U*^13^	*U*^23^
C1	0.0227 (11)	0.0238 (17)	0.0172 (9)	0.0024 (11)	0.0065 (8)	0.0079 (9)
C2	0.022 (4)	0.026 (4)	0.022 (3)	−0.002 (3)	0.004 (3)	0.006 (3)
C3	0.024 (3)	0.030 (3)	0.027 (4)	0.001 (2)	0.006 (3)	0.005 (3)
C4	0.032 (5)	0.046 (4)	0.025 (4)	−0.004 (3)	0.017 (3)	0.001 (3)
C5	0.040 (5)	0.059 (7)	0.024 (4)	0.000 (4)	0.009 (3)	0.022 (4)
C6	0.030 (4)	0.033 (3)	0.029 (3)	0.005 (3)	0.012 (3)	0.015 (2)
C1B	0.0227 (11)	0.0238 (17)	0.0172 (9)	0.0024 (11)	0.0065 (8)	0.0079 (9)
C2B	0.022 (5)	0.020 (5)	0.016 (4)	0.005 (5)	0.004 (3)	0.006 (3)
C3B	0.036 (6)	0.034 (5)	0.018 (5)	0.015 (4)	0.016 (4)	0.012 (4)
C4B	0.027 (5)	0.026 (4)	0.015 (3)	0.001 (4)	0.007 (4)	0.009 (3)
C5B	0.033 (5)	0.023 (5)	0.012 (4)	0.008 (4)	0.007 (3)	0.006 (4)
C6B	0.023 (5)	0.026 (4)	0.016 (3)	0.003 (3)	0.002 (4)	0.010 (3)
C7	0.0291 (10)	0.0221 (9)	0.0232 (9)	0.0069 (8)	0.0079 (8)	0.0102 (8)
C8	0.0487 (13)	0.0264 (10)	0.0347 (11)	0.0097 (9)	0.0222 (10)	0.0158 (9)
C9	0.0496 (14)	0.0271 (10)	0.0358 (12)	0.0089 (9)	0.0148 (10)	0.0187 (9)
C10	0.0481 (14)	0.0232 (10)	0.0356 (11)	0.0112 (9)	0.0138 (10)	0.0130 (9)
C11	0.0446 (13)	0.0268 (10)	0.0399 (12)	0.0117 (9)	0.0209 (11)	0.0106 (9)
C12	0.0359 (11)	0.0230 (9)	0.0298 (10)	0.0055 (8)	0.0158 (9)	0.0098 (8)
C13	0.023 (4)	0.013 (2)	0.013 (3)	0.0053 (19)	0.002 (2)	0.007 (2)
C14	0.024 (2)	0.029 (2)	0.028 (3)	0.0036 (15)	0.007 (2)	0.017 (2)
C15	0.033 (4)	0.026 (4)	0.054 (3)	0.008 (3)	0.009 (3)	0.019 (3)
C16	0.027 (2)	0.031 (2)	0.034 (4)	−0.0029 (17)	0.008 (3)	0.016 (2)
C17	0.025 (5)	0.025 (4)	0.043 (4)	0.003 (3)	0.013 (3)	0.019 (3)
C18	0.019 (4)	0.020 (5)	0.031 (4)	−0.004 (3)	0.008 (3)	0.011 (3)
C13B	0.020 (5)	0.033 (8)	0.021 (6)	−0.005 (4)	0.010 (4)	0.004 (4)
C14B	0.034 (3)	0.026 (3)	0.044 (5)	0.007 (2)	0.018 (4)	0.015 (4)
C15B	0.011 (5)	0.063 (9)	0.065 (8)	0.003 (4)	0.011 (4)	0.034 (5)
C16B	0.036 (4)	0.036 (4)	0.029 (5)	−0.003 (3)	0.014 (4)	0.012 (4)
C17B	0.043 (9)	0.023 (6)	0.039 (7)	0.001 (5)	0.015 (5)	0.011 (4)
C18B	0.041 (9)	0.031 (8)	0.034 (7)	0.020 (6)	0.017 (6)	0.020 (6)
P1	0.0191 (2)	0.0180 (2)	0.0148 (2)	0.00359 (17)	0.00545 (18)	0.00827 (17)
Cl1	0.0231 (2)	0.0357 (2)	0.0193 (2)	0.00003 (18)	0.00788 (18)	0.01451 (19)
Pd1	0.01700 (11)	0.01711 (11)	0.01244 (10)	0.00288 (7)	0.00508 (7)	0.00760 (7)

### Geometric parameters (Å, º)

**Table:**

C1—C2	1.3900	C10—H10A	0.9700
C1—C6	1.3900	C10—H10B	0.9700
C1—P1	1.825 (7)	C11—C12	1.527 (3)
C2—C3	1.3900	C11—H11A	0.9700
C2—H2	0.9300	C11—H11B	0.9700
C3—C4	1.3900	C12—H12A	0.9700
C3—H3	0.9300	C12—H12B	0.9700
C4—C5	1.3900	C13—C14	1.528 (8)
C4—H4	0.9300	C13—C18	1.531 (9)
C5—C6	1.3900	C13—P1	1.813 (7)
C5—H5	0.9300	C13—H13	0.9800
C6—H6	0.9300	C14—C15	1.523 (8)
C1B—C6B	1.524 (10)	C14—H14A	0.9700
C1B—C2B	1.534 (9)	C14—H14B	0.9700
C1B—P1	1.843 (10)	C15—C16	1.519 (7)
C1B—H1B	0.9800	C15—H15A	0.9700
C2B—C3B	1.538 (11)	C15—H15B	0.9700
C2B—H2BA	0.9700	C16—C17	1.516 (10)
C2B—H2BB	0.9700	C16—H16A	0.9700
C3B—C4B	1.491 (12)	C16—H16B	0.9700
C3B—H3BA	0.9700	C17—C18	1.517 (8)
C3B—H3BB	0.9700	C17—H17A	0.9700
C4B—C5B	1.503 (10)	C17—H17B	0.9700
C4B—H4BA	0.9700	C18—H18A	0.9700
C4B—H4BB	0.9700	C18—H18B	0.9700
C5B—C6B	1.536 (9)	C13B—C14B	1.3900
C5B—H5BA	0.9700	C13B—C18B	1.3900
C5B—H5BB	0.9700	C13B—P1	1.891 (7)
C6B—H6BA	0.9700	C14B—C15B	1.3900
C6B—H6BB	0.9700	C14B—H14C	0.9300
C7—C8	1.520 (3)	C15B—C16B	1.3900
C7—C12	1.528 (3)	C15B—H15C	0.9300
C7—P1	1.8421 (19)	C16B—C17B	1.3900
C7—H7	0.9800	C16B—H16C	0.9300
C8—C9	1.524 (3)	C17B—C18B	1.3900
C8—H8A	0.9700	C17B—H17C	0.9300
C8—H8B	0.9700	C18B—H18C	0.9300
C9—C10	1.516 (3)	P1—Pd1	2.3343 (5)
C9—H9A	0.9700	Cl1—Pd1	2.3017 (4)
C9—H9B	0.9700	Pd1—Cl1^i^	2.3017 (4)
C10—C11	1.507 (3)	Pd1—P1^i^	2.3343 (5)
			
C2—C1—C6	120.0	C12—C11—H11B	109.2
C2—C1—P1	117.9 (5)	H11A—C11—H11B	107.9
C6—C1—P1	121.8 (5)	C11—C12—C7	110.85 (17)
C1—C2—C3	120.0	C11—C12—H12A	109.5
C1—C2—H2	120.0	C7—C12—H12A	109.5
C3—C2—H2	120.0	C11—C12—H12B	109.5
C4—C3—C2	120.0	C7—C12—H12B	109.5
C4—C3—H3	120.0	H12A—C12—H12B	108.1
C2—C3—H3	120.0	C14—C13—C18	109.4 (6)
C3—C4—C5	120.0	C14—C13—P1	114.9 (5)
C3—C4—H4	120.0	C18—C13—P1	111.5 (5)
C5—C4—H4	120.0	C14—C13—H13	106.9
C6—C5—C4	120.0	C18—C13—H13	106.9
C6—C5—H5	120.0	P1—C13—H13	106.9
C4—C5—H5	120.0	C15—C14—C13	110.9 (6)
C5—C6—C1	120.0	C15—C14—H14A	109.5
C5—C6—H6	120.0	C13—C14—H14A	109.5
C1—C6—H6	120.0	C15—C14—H14B	109.5
C6B—C1B—C2B	109.7 (7)	C13—C14—H14B	109.5
C6B—C1B—P1	114.9 (8)	H14A—C14—H14B	108.0
C2B—C1B—P1	113.6 (8)	C16—C15—C14	111.4 (5)
C6B—C1B—H1B	106.0	C16—C15—H15A	109.4
C2B—C1B—H1B	106.0	C14—C15—H15A	109.4
P1—C1B—H1B	106.0	C16—C15—H15B	109.4
C1B—C2B—C3B	110.2 (6)	C14—C15—H15B	109.4
C1B—C2B—H2BA	109.6	H15A—C15—H15B	108.0
C3B—C2B—H2BA	109.6	C17—C16—C15	110.2 (6)
C1B—C2B—H2BB	109.6	C17—C16—H16A	109.6
C3B—C2B—H2BB	109.6	C15—C16—H16A	109.6
H2BA—C2B—H2BB	108.1	C17—C16—H16B	109.6
C4B—C3B—C2B	111.5 (7)	C15—C16—H16B	109.6
C4B—C3B—H3BA	109.3	H16A—C16—H16B	108.1
C2B—C3B—H3BA	109.3	C16—C17—C18	112.1 (6)
C4B—C3B—H3BB	109.3	C16—C17—H17A	109.2
C2B—C3B—H3BB	109.3	C18—C17—H17A	109.2
H3BA—C3B—H3BB	108.0	C16—C17—H17B	109.2
C3B—C4B—C5B	111.3 (8)	C18—C17—H17B	109.2
C3B—C4B—H4BA	109.4	H17A—C17—H17B	107.9
C5B—C4B—H4BA	109.4	C17—C18—C13	110.4 (6)
C3B—C4B—H4BB	109.4	C17—C18—H18A	109.6
C5B—C4B—H4BB	109.4	C13—C18—H18A	109.6
H4BA—C4B—H4BB	108.0	C17—C18—H18B	109.6
C4B—C5B—C6B	110.8 (7)	C13—C18—H18B	109.6
C4B—C5B—H5BA	109.5	H18A—C18—H18B	108.1
C6B—C5B—H5BA	109.5	C14B—C13B—C18B	120.0
C4B—C5B—H5BB	109.5	C14B—C13B—P1	121.5 (5)
C6B—C5B—H5BB	109.5	C18B—C13B—P1	118.4 (5)
H5BA—C5B—H5BB	108.1	C15B—C14B—C13B	120.0
C1B—C6B—C5B	109.8 (8)	C15B—C14B—H14C	120.0
C1B—C6B—H6BA	109.7	C13B—C14B—H14C	120.0
C5B—C6B—H6BA	109.7	C16B—C15B—C14B	120.0
C1B—C6B—H6BB	109.7	C16B—C15B—H15C	120.0
C5B—C6B—H6BB	109.7	C14B—C15B—H15C	120.0
H6BA—C6B—H6BB	108.2	C15B—C16B—C17B	120.0
C8—C7—C12	111.13 (16)	C15B—C16B—H16C	120.0
C8—C7—P1	113.28 (14)	C17B—C16B—H16C	120.0
C12—C7—P1	111.08 (13)	C18B—C17B—C16B	120.0
C8—C7—H7	107.0	C18B—C17B—H17C	120.0
C12—C7—H7	107.0	C16B—C17B—H17C	120.0
P1—C7—H7	107.0	C17B—C18B—C13B	120.0
C7—C8—C9	110.74 (18)	C17B—C18B—H18C	120.0
C7—C8—H8A	109.5	C13B—C18B—H18C	120.0
C9—C8—H8A	109.5	C13—P1—C1	104.6 (6)
C7—C8—H8B	109.5	C13—P1—C7	103.3 (3)
C9—C8—H8B	109.5	C1—P1—C7	107.4 (3)
H8A—C8—H8B	108.1	C13—P1—C1B	108.0 (6)
C10—C9—C8	111.03 (17)	C7—P1—C1B	103.5 (5)
C10—C9—H9A	109.4	C1—P1—C13B	98.7 (5)
C8—C9—H9A	109.4	C7—P1—C13B	106.9 (3)
C10—C9—H9B	109.4	C1B—P1—C13B	102.1 (7)
C8—C9—H9B	109.4	C13—P1—Pd1	115.0 (3)
H9A—C9—H9B	108.0	C1—P1—Pd1	114.7 (3)
C11—C10—C9	111.58 (18)	C7—P1—Pd1	110.95 (6)
C11—C10—H10A	109.3	C1B—P1—Pd1	114.8 (4)
C9—C10—H10A	109.3	C13B—P1—Pd1	117.3 (4)
C11—C10—H10B	109.3	Cl1—Pd1—Cl1^i^	180.0
C9—C10—H10B	109.3	Cl1—Pd1—P1	89.296 (16)
H10A—C10—H10B	108.0	Cl1^i^—Pd1—P1	90.704 (16)
C10—C11—C12	112.03 (18)	Cl1—Pd1—P1^i^	90.704 (16)
C10—C11—H11A	109.2	Cl1^i^—Pd1—P1^i^	89.296 (16)
C12—C11—H11A	109.2	P1—Pd1—P1^i^	180.0
C10—C11—H11B	109.2		

Symmetry code: (i) −*x*+2, −*y*, −*z*+2.

### Hydrogen-bond geometry (Å, º)

**Table:**

*D*—H···*A*	*D*—H	H···*A*	*D*···*A*	*D*—H···*A*
C8—H8*B*···Cl1	0.97	2.91	3.559 (2)	125
C5—H5···Cl1^ii^	0.93	2.94	3.619 (7)	131
C13—H13···Cl1^i^	0.98	2.68	3.254 (11)	118
C18—H18*B*···Cl1^i^	0.97	2.97	3.542 (13)	119
C15—H15*A*···Cl1^iii^	0.97	3.02	3.800 (8)	139

Symmetry codes: (i) −*x*+2, −*y*, −*z*+2; (ii) −*x*+2, −*y*, −*z*+1; (iii) *x*−1, *y*, *z*.
